# Theta- and Gamma-Band Activity Discriminates Face, Body and Object Perception

**DOI:** 10.3389/fnhum.2020.00074

**Published:** 2020-03-12

**Authors:** Francesco Bossi, Isabella Premoli, Sara Pizzamiglio, Sema Balaban, Paola Ricciardelli, Davide Rivolta

**Affiliations:** ^1^Department of Psychology, University of Milan—Bicocca, Milan, Italy; ^2^School of Psychology, University of East London (UEL), London, United Kingdom; ^3^Institute of Psychiatry, Psychology, and Neuroscience (IoPPN), King’s College London, London, United Kingdom; ^4^School of Architecture, Computing and Engineering, University of East London (UEL), London, United Kingdom; ^5^NeuroMI: Milan Center for Neuroscience, Milan, Italy; ^6^Department of Education, Psychology, and Communication, University of Bari Aldo Moro, Bari, Italy

**Keywords:** neural oscillations, face-inversion effect, body-inversion effect, gamma activity, theta activity, configural processing

## Abstract

Face and body perception is mediated by configural mechanisms, which allow the perception of these stimuli as a whole, rather than the sum of individual parts. Indirect measures of configural processing in visual cognition are the face and body inversion effects (FIE and BIE), which refer to the drop in performance when these stimuli are perceived upside-down. Albeit FIE and BIE have been well characterized at the behavioral level, much still needs to be understood in terms of the neurophysiological correlates of these effects. Thus, in the current study, the brain’s electrical activity has been recorded by a 128 channel electroencephalogram (EEG) in 24 healthy participants while perceiving (upright and inverted) faces, bodies and houses. EEG data were analyzed in both the time domain (i.e., event-related potentials—ERPs) and the frequency domain [i.e., induced theta (5–7 Hz) and gamma (28–45 Hz) oscillations]. ERPs amplitude results showed increased N170 amplitude for inverted faces and bodies (compared to the same stimuli presented in canonical position) but not for houses. ERPs latency results showed delayed N170 components for inverted (vs. upright) faces, houses, but not bodies. Spectral analysis of induced oscillations indicated physiological FIE and BIE; that is decreased gamma-band synchronization over right occipito-temporal electrodes for inverted (vs. upright) faces, and increased bilateral frontoparietal theta-band synchronization for inverted (vs. upright) faces. Furthermore, increased left occipito-temporal and right frontal theta-band synchronization for upright (vs. inverted) bodies was found. Our findings, thus, demonstrate clear differences in the neurophysiological correlates of face and body perception. The neurophysiological FIE suggests disruption of feature binding processes (decrease in occipital gamma oscillations for inverted faces), together with enhanced feature-based attention (increase in frontoparietal theta oscillations for inverted faces). In contrast, the BIE may suggest that structural encoding for bodies is mediated by the first stages of configural processing (decrease in occipital theta oscillations for inverted bodies).

## Introduction

Humans can identify hundreds of faces with ease, although all share a common 3D structure (i.e., two eyes above the nose, which is in turn above the mouth). It is believed that this extraordinary ability is mediated by face-sensitive perceptual mechanisms (i.e., configural processing), which allow the perception of faces as wholes (i.e., as *gestalts*), rather than a sum of the individual components (McKone and Yovel, 2009; Monti et al., 2020). The reduced accuracy (and increased latency) in recognizing faces when they are perceived upside-down rather than in their canonical orientation is known as the “face inversion effect” (FIE; Yin, 1969), and has traditionally been considered as (indirect) evidence for the existence of configural processing for upright faces only. In addition, since this effect is much smaller for non-face objects (Valentine, 1988) and objects of expertise (Robbins and McKone, 2007), it has been suggested that FIE might be face-specific, thus leading to the conclusion that configural processing *only* mediates upright face perception (*ibidem*).

By *configural processing*, we refer to any phenomenon that involves perceiving spatial relations (i.e., configuration) among the features of a stimulus, such as a face (Reed et al., 2006). Maurer et al. (2002) identified distinct stages of configural processing: (i) *first-order spatial relations* define the relative positions in space of the parts of an object, such as the placement of the eyes above the nose; (ii) *second-order relational information* that refers to the exact metric distances between parts, e.g., the distance between eyes, nose, and mouth; and (iii) the last stage of configural processing is represented by the *holistic* stage or undifferentiated template representation of the face (i.e., perceiving the face as a whole; Gauthier and Tarr, 2002). Inversion was proven to affect all these configural processing stages (Maurer et al., 2002), and face processing is thought to specifically require and rely on the last stage (i.e., the holistic stage; *ibidem*).

Electrophysiological markers of holistic processing have been reported by means of electroencephalography (EEG), a technique that monitors the brain’s electrical activity with excellent temporal resolution (Tucker, 1993). Much evidence suggests the existence of a face-sensitive event-related potential (ERP) negative component peaking at around 170 ms post-stimulus onset (N170) and reflecting early perceptual processing of the human visual system (Bentin et al., 1996, 1999; Rossion and Gauthier, 2002). Given that the N170 is larger and delayed for inverted faces, it is believed that this component reflects early visual structural encoding (Rossion et al., 2000; Watanabe et al., 2003). These findings have also been corroborated using magnetoencephalography (MEG), which allows the recording of neuromagnetic activity (Rivolta et al., 2012, 2014).

ERPs reflect brain activity that is phase-locked (i.e., evoked) to the stimulus onset, and they are calculated by averaging the EEG signal from all trials. However, ERPs hide information derived from induced (non-phase locked) activity, which mainly reflects high-cognitive, rather than perceptual, activity (Uhlhaas and Singer, 2010; Donner and Siegel, 2011). The induced activity can be extracted on a single-trial level and can be retrieved by time-frequency analyses, also known as time-frequency representations (TFRs), on different frequency bands (Donner and Siegel, 2011; Oostenveld et al., 2011; Rivolta et al., 2015).

In the visual system, high-frequency, low amplitude gamma-band (>25 Hz) activity has been suggested to mediate perceptual binding and the grouping of visual information (Singer and Gray, 1995; Tallon-Baudry and Bertrand, 1999; Grent-t’-Jong et al., 2016). According to the “representational hypothesis,” induced gamma synchronization is a sign of visual features binding, also related to the holistic processing of faces (Tallon-Baudry and Bertrand, 1999). In response to coherent visual stimuli (e.g., faces, as well as objects perceived in visual illusions), the induced gamma activity was focused at occipital and parieto-occipital locations, suggesting that it originates, at least in part, in visual areas (Grützner et al., 2013). This hypothesis is also supported by the finding that it partially follows a retinotopic organization. This definition implies that gamma-band oscillations represent a marker of holistic processing and second-order spatial information processing (i.e., the highest stages of configural processing; Maurer et al., 2002). Evidence suggests that enhanced gamma oscillations are induced by faces over occipito-temporal areas when compared to control stimuli such as houses or scrambled stimuli (Zion-Golumbic and Bentin, 2007; Zion-Golumbic et al., 2008; Gao et al., 2012). Since these oscillations show a physiological FIE (i.e., upright faces induced enhanced synchronization in gamma-band oscillations when compared to inverted faces), this activity likely reflects the difficulty of the visual system to bind facial features of inverted faces (i.e., holistic processing is not engaged by inverted stimuli) in a configural representation (Lachaux et al., 2005; Anaki et al., 2007; Dobel et al., 2011; Moratti et al., 2014; Matsuzaki et al., 2015; Uono et al., 2017). In line with behavioral results, even non-face stimuli (i.e., objects) show a physiological inversion effect, albeit of smaller magnitude (Tallon-Baudry and Bertrand, 1999). Overall, behavioral and physiological (EEG/MEG) data converge and indicate that upright face perception is mediated by holistic processing, which, in turn, is mediated by gamma-band synchronization in the visual system.

Lower frequency oscillations, especially in the theta-band (4–7 Hz), correlate with various cognitive and attentional mechanisms (Ptak et al., 2017). It has been shown that cortico-hippocampal interactions mediate theta activity in cognition (Lopes da Silva, 1992; Başar, 1999) since theta activity represents the spontaneous rhythm of different limbic structures. Although theta-band connections between limbic structures and the visual cortex are not specifically linked to the FIE, they have been reported in various emotional paradigms (Aftanas et al., 2001, 2002) and several experiments involving facial recognition and facial emotional expressions (Başar et al., 2006; Güntekin and Başar, 2009; Güntekin and Başar, 2014). In those studies, occipital and occipito-temporal areas showed enhanced theta synchronization when processing emotionally arousing visual stimuli or faces showing emotional expressions at latencies around 200–500 ms post-stimulus. Theta synchronization was also observed when processing emotional facial expressions over different regions (Balconi and Lucchiari, 2006; Knyazev et al., 2009; Zhang et al., 2012). Furthermore, enhanced theta-gamma coupling induced by upright (as compared to inverted) faces has been shown in the right inferior occipital gyrus (IOG) after 200 ms post-stimulus onset (Sato et al., 2014, 2017). These findings suggest that occipital theta-band oscillations may represent a marker of the fast, early, perceptual processing of highly *salient* stimuli.

Albeit critical in human social interactions, faces do not represent the only stimuli we rely on; recognition of individuals also relies heavily on body processing. Similar to faces, bodies constitute fundamental mediums for emotional expression and communication, and they show a universal configuration (i.e., torso, arms, legs; for a review, see de Gelder et al., 2015). One of the main differences between the two categories is that, while faces are a very important medium to convey identity, bodies strongly convey information about actions and intentions (Iacoboni et al., 2005). At the cognitive and neurophysiological level, however, body and face processing share certain perceptual mechanisms: body inversion, like faces, causes a drop in performance (i.e., the body inversion effect, BIE; Reed et al., 2003; Bonemei et al., 2018), which suggests that even body perception is mediated by configural processing. Additionally, as for faces, the N170 is larger and delayed for inverted bodies (Stekelenburg and de Gelder, 2004). When considering configural body processing, Reed et al. (2006) identified another stage of configural processing, located in between first- and second-order relational information: *structural information* or *hierarchical structure stage*. Structural information refers to information about the organization of parts in terms of the overall object as well as the spatial relationship of each type of part relative to each other. For instance, in a body, arms and legs may vary in how far above or below each other are in space, but they are still connected to the same regions of the torso, which defines the overall hierarchical structure of the body. Even though both face and body perception relies on configural processing, these categories may involve different perceptual stages to different extents (Reed et al., 2006): while face processing relies on all levels of configural processing (Maurer et al., 2002), the study by Reed et al. (2006) showed that body processing seems to rely only on lower stages of configural processing (i.e., first-order relational information and structural information) and not on higher stages (i.e., holistic processing and second-order relational information; see also Minnebusch and Daum, 2009). Indeed, they found a BIE in posture recognition when inverting intact bodies and half body pictures divided along the vertical axis (maintaining structural information), but not half body pictures divided along the horizontal axis (losing structural information), single body parts (based on feature processing) or scrambled bodies (losing first-order relational information; Reed et al., 2006).

Although ERPs of the BIE has been investigated, no study has so far compared oscillatory activity for face and body perception. Thus, in the current study, we compared neural oscillations elicited by the presentation of upright and inverted faces to those elicited by (upright and inverted) bodies and houses. Houses were chosen as control stimuli since, like bodies and faces, they can vary in specific features and their configuration, but they are not social stimuli (Negrini et al., 2017).

We focused our attention on gamma- and theta-band activity; the former is a specific marker of feature binding processes (Tallon-Baudry and Bertrand, 1999) and holistic processing (Bentin et al., 1996, 1999; Rossion et al., 2000), whereas the latter is involved in the fast early perceptual processing of salient stimuli (Başar et al., 2006; Güntekin and Başar, 2009; Güntekin and Başar, 2014). We, thus, hypothesized to observe greater gamma-band activity when participants were processing upright rather than inverted faces. In addition, based on the previous literature showing the importance of theta- and gamma-band activity in feature binding and facial recognition processes (Tallon-Baudry and Bertrand, 1999; Güntekin and Başar, 2014), we also expected to find some differences in processing between our stimuli categories in theta and gamma activity. More specifically, if the perceptual processes disrupted by face and body inversion were the same, then a similar pattern of oscillatory activity should be elicited by the inversion of both categories of visual stimuli, while a different pattern should be found for inverted houses. In contrast, if this was not the case, we expected to find different oscillatory patterns for each of the three stimulus categories.

## Materials and Methods

### Participants

Twenty-four healthy participants (11 M; mean age: 28.2 ± 5.8 years), mainly among university students and their acquaintances, were recruited. One participant was excluded from the analyses due to technical problems related to data quality. All participants gave written informed consent before enrolment in this study and were screened for contraindications to EEG: exclusion criteria included the presence of a history of any neurological or psychiatric disease, use of active drugs, abuse of any drugs (including nicotine and alcohol) as well as any skin condition that could be worsened by the use of the EEG cap. The study was approved by the local Ethics Committee of the University of East London (UEL) and was conducted in accordance with the ethical standards laid out in the 1964 Declaration of Helsinki. All participants had a normal or corrected-to-normal vision and were right-handed.

### Stimuli

A total of 96 pictures were presented to each participant (one per trial). Thirty-two pictures of faces were extracted from the Radboud Faces Database (RaFD; Langner et al., 2010), 32 pictures of bodies were extracted from the Bodily Expressive Action Stimulus Test (BEAST; de Gelder and Van den Stock, 2011), and 32 pictures of houses were extracted from the dataset used in a previous EEG experiment (Negrini et al., 2017). All pictures representing faces and bodies conveyed neutral expression, and they depicted 32 different actors for faces and 32 for bodies (balanced for gender). Half of the pictures were presented upright, and the other half were inverted, in a counterbalanced design across participants.

All pictures were converted into black and white images and cropped to a blank background using Adobe Photoshop CS5 software (Adobe Systems, Inc, 2011) with a dimension of 7 × 10.5 cm, which subtended a visual angle of 4° × 6° on a 22-inch LCD monitor positioned 100 cm away from participants. To match all stimuli’s low-level visual features, mean luminance was manipulated and balanced using MATLAB^®^ R2016a (Mathworks, Inc, 2016) and the SHINE toolbox (Willenbockel et al., 2010) by means of a customized script.

### Procedure

After participants gave written informed consent, the EEG cap was put on (see next section for details). Participants were conducted into and seated in a dimly illuminated electrically shielded room, where the cap was connected to the EEG amplifier, and participants began the experiment. The experiment was run using E-Prime^®^ 2.0 software (Schneider et al., 2007). Participants were presented with 96 stimuli that were divided into three blocks of 32 stimuli and randomly presented using a permutated block order for each participant. Before each block, five trials were presented as practice, and feedback was given on the participant’s response. Each trial consisted of a fixation cross shown for 1 s and a stimulus displayed for 500 ms, followed by a response screen (maximum duration: 5 s), during which the participant could respond. Participants had to judge whether the stimulus was presented *upright* or *inverted* by pressing one of two buttons on an EGI^®^ Chronos response box. After the response (or after 5 s of response screen presentation), a gray screen was presented for 1 s before the next trial began.

### EEG Data Recording and Analysis

EEG data were recorded using a high-density 128-channel Hydrocel Geodesic Sensor Net (Electrical Geodesic Inc., EGI, Eugene, OR, USA) referenced to the vertex (Tucker, 1993). The EEG signal was amplified with an EGI NetAmps 400 amplifier, digitized at a 1,000 Hz sampling rate, and recorded. No filters were applied during signal recording. Electrode impedances were kept below 50 kΩ throughout the experimental procedure.

EEG data were analyzed using MATLAB^®^ version R2016a (Mathworks, Inc, 2016) and customized scripts as well as the EEGLAB (Delorme and Makeig, 2004) and FieldTrip toolboxes (Oostenveld et al., 2011). A band-pass filter (1–100 Hz) and a notch filter (50 Hz) were applied to limit the signal of interest and remove power line noise. Data were subsequently segmented into epochs (i.e., trials) of 2,000 ms length, starting from the presentation of the fixation cross and ending 500 ms after presentation of the response screen. Each trial was baseline-corrected by removing the values averaged over a period of 1,000 ms (from 1,000 to 0 ms before the stimulus), during which participants were looking at the fixation cross. After visual inspection, trials affected by prominent artifacts (i.e., major muscle movement and electric artifacts) were removed, and bad channels were deleted. On average, 90 trials per participant were included in the analysis. The signal was referenced to the common average of all electrodes (Dien, 1998), and independent component analysis (ICA) was applied to remove the remaining artifacts related to the muscular and ocular activity. After we removed the remaining artifacts using ICA, noisy channels were spatially interpolated.

**Figure 1:**
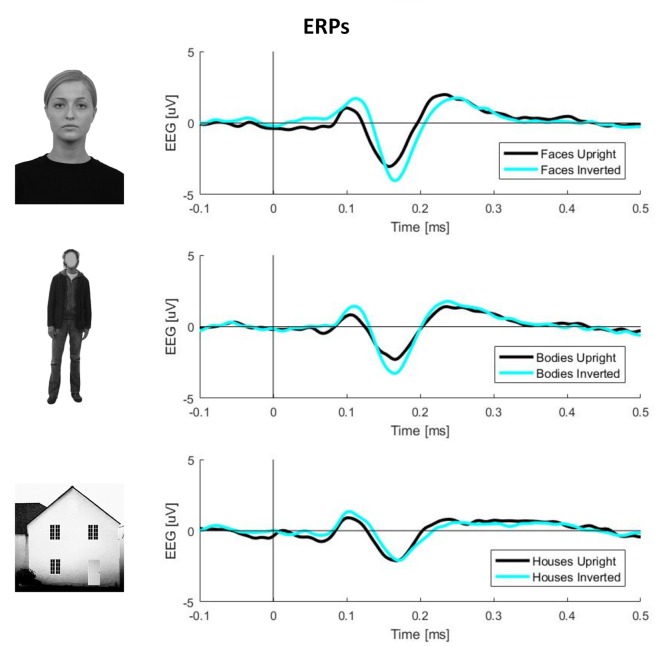
Plots of event-related potential (ERP) activity calculated over 11 right occipito-temporal electrodes, averaged over 23 participants separately for three stimulus categories (faces, bodies, and houses). In each plot, the black line represents upright stimuli, while the cyan line represents inverted stimuli. This picture clearly shows larger N170 components (negative deflections around 170 ms post-stimulus onset) for inverted faces and bodies but not houses.

To obtain ERPs, all trials were averaged for each condition and participant. The N170 component amplitude was computed by averaging the activity in the range of 140–200 ms. The exact time-window was defined by visual inspection of the butterfly plots for each condition (Figure 1).

**Figure 2:**
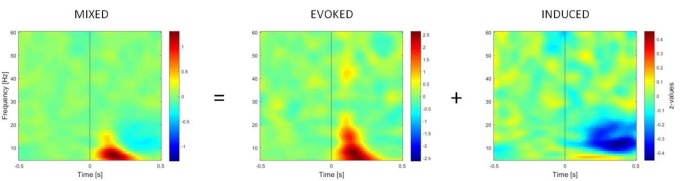
Time-frequency representations (TFRs) of mixed, evoked (phase-locked) and induced (non-phase-locked) activity, calculated over 11 right occipito-temporal electrodes (region of interest determined by the literature on the face inversion effect), averaged over 23 participants. Activity elicited by upright faces is shown. The picture shows how the induced activity is determined by computing the TFR of evoked activity (on ERPs averaged across trials) and subtracting it from the mixed activity at the single-trial level.

TFRs of oscillatory power changes were computed separately for each of the six stimulus categories (faces upright and inverted, bodies upright and inverted, houses upright and inverted). Time-frequency power spectra were estimated using Morlet wavelet analysis based on 3.5 cycles at the lowest frequency (5 Hz) increasing to 18 cycles at the highest considered frequency (60 Hz; time steps: 10 ms; frequency steps: 1 Hz; Oostenveld et al., 2011). We divided neuronal response components into those evoked (i.e., phase-locked) vs. induced (i.e., non-phase-locked) by stimuli (Figure 2; David et al., 2006; Donner and Siegel, 2011; Cohen and Donner, 2013; Herrmann et al., 2014). The TFR of the induced response was then isolated by subtracting the individual time-domain average from each trial before calculating the TFRs for single trials (Cohen and Donner, 2013; Premoli et al., 2017). This approach was adopted since we performed single-trial normalization by z-transforming the TFR of each trial for each frequency. The z-transformation was performed on the respective mean and standard deviation derived from the full trial length. Following the z-transformation, an absolute baseline correction for each trial was performed by subtracting the average of the −400 to −100 ms period for each frequency to ensure z-values represented a change from the baseline (Premoli et al., 2017). The baseline correction time-window (−400 to −100 ms) was chosen to avoid evoked time-frequency activity that could be found some ms before stimulus onset in low frequencies. Subsequently, TFRs were averaged across trials per experimental condition. After performing this procedure, the result consisted of an event-related spectral perturbation (ERSP) measure that is robustly normalized based on the single-trial level (Grandchamp and Delorme, 2011). In the end, TFRs were cropped to the period of interest (−500 to 500 ms), removing time-frequency bins at the trial edges for which no values could be computed. Values were averaged across frequency bins to calculate power within two frequency bands, namely theta (5–7 Hz) and gamma (28–45 Hz), which are considered to be the most representative frequency ranges in the study of social visual stimuli (Güntekin and Başar, 2014).

The gamma range limits were chosen since phase-locked, and time-locked gamma oscillations following visual stimulation are detectable in the 28–45 Hz range in different time-windows (Başar, 2012).

### Statistical Analyses

To compare sensor-level EEG data between stimulus conditions, non-parametric cluster-based permutation analyses (using a Monte-Carlo method based on paired *t*-statistics) were performed (Maris and Oostenveld, 2007). This method was shown to be very accurate in solving the multiple comparisons problem in M/EEG data, and it has been compared with other broadly used approaches (i.e., bootstrap-based and Bayesian approaches; Maris, 2012). Considering data separated by frequency range and time-window, *t*-values exceeding an *a priori* threshold of *p* < 0.05 were clustered based on neighboring electrodes. Cluster-level statistics were calculated by taking the sum of the *t*-values within every cluster. Comparisons were performed for the maximum values of summed *t*-values. Using a permutation test (i.e., randomizing data across conditions and re-running the statistical test 1,500 times), we obtained a reference distribution of the maximum of summed cluster-level *t*-values to evaluate the statistic from the actual data. Clusters in the dataset were considered statistically significant at an alpha level of 0.05 if <5% of the permutations (*N* = 1,500) used to construct the reference distribution yielded a maximum cluster-level statistic larger than the cluster-level value observed in the original data.

To test whether our data replicated previous findings, three paired-samples *t*-tests were performed separately on ERPs computed by averaging trials for each participant in each condition. These comparisons investigated the inversion effect in different categories (faces upright vs. inverted, bodies upright vs. inverted, and houses upright vs. inverted) on the N170 component amplitude. Successively, ERP differences between upright and inverted stimuli were computed for the three categories (faces, bodies, and houses) by subtracting averaged μV values in the inverted condition from those in the upright condition for each timepoint. These differences were then compared by performing three paired-samples *t*-tests to test any interaction effects (face inversion vs. body inversion, face inversion vs. house inversion, and body inversion vs. house inversion).

To investigate FIE and BIE on N170 more in-depth, we also performed a repeated-measure ANOVA on N170 latency. The downside of this approach consists of an a-priori selection of channels, which is not necessary for cluster-based tests. For this reason, we selected only channels presenting a statistically significant difference in amplitude tests for FIE. Peak latency from these 34 resulting occipitotemporal channels was averaged and analyzed using inversion (upright vs. inverted) and stimulus (faces vs. bodies vs. houses) as independent variables.

**Figure 3:**
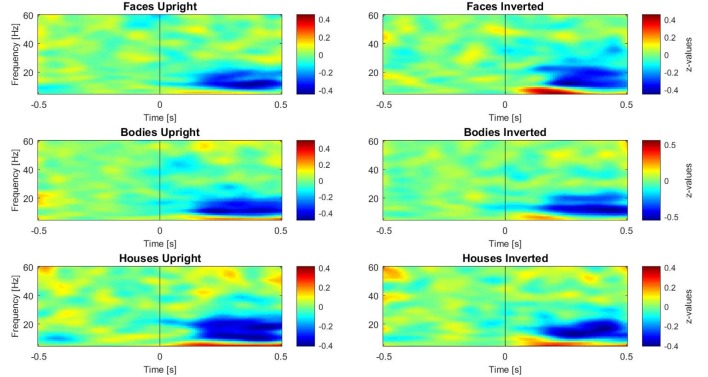
Plots of induced time-frequency activity calculated over 11 right occipito-temporal electrodes and averaged over 23 participants for each category of stimuli. This picture allows the reader to observe differences in patterns of activity for each category, in particular over the theta (5–7 Hz) and gamma (28–45 Hz) bands.

Subsequently, three paired-samples *t*-tests were performed separately on induced data to investigate the inversion effect in different category comparisons (faces upright vs. inverted, bodies upright vs. inverted, and houses upright vs. inverted). For these tests, the activity in different frequency ranges was separated as described above. One time-window of interest (TOI) was defined by both referring to the existing literature on face perception and visual inspection of occipital single plots of activity (Figure 3): the epoch of interest for induced activity was set to 250–500 ms. This TOI was chosen because induced activity (especially in the gamma range) typically starts approximately 280 ms after presentation of the stimulus and is clearly disentangled from evoked activity at this latency (Tallon-Baudry and Bertrand, 1999), while 500 ms was chosen as the limit of the TOI because it is the time when the response screen was presented; thus, we expected motor-related activity after this time-window. In this time-window, z-transformed values were averaged across time bins for each frequency.

Behavioral data were not analyzed for this study since participants’ task was extremely basic (identifying stimulus orientation, upright vs. inverted). Indeed, participants’ accuracy reached a ceiling effect (mean accuracy = 0.98). The task did not require to process information that could be harder to acquire when structural encoding is disrupted. For this reason, we did not expect to find a behavioral inversion effect on accuracy or response times: structural encoding is necessary to perform the task (identify whether the stimuli are presented upright or inverted), but would not influence participants’ behavioral responses.

## Results

### ERP Analysis

#### N170 Amplitude

**Figure 4:**
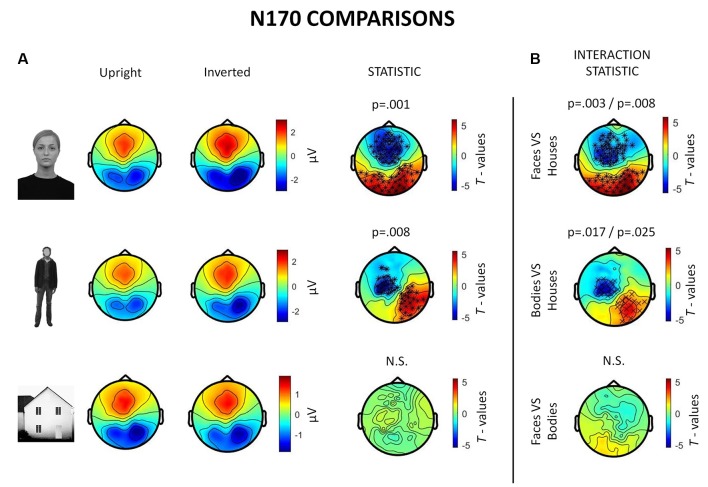
Summary of ERP results. All topographies were obtained by calculating the average voltage over the time-window of interest (TOI) for N170 components (i.e., 140–200 ms after stimulus onset). **(A)** Topographies show the activity evoked by upright and inverted faces (first row), bodies (second row) and houses (third row). The third column of topographies shows clusters where statistically significant differences between upright and inverted stimuli were found by means of non-parametric cluster-based permutation tests. Inverted faces and bodies showed a larger N170 (occipito-temporal areas) and VPP (vertex positive potential, frontal areas) than upright stimuli. **(B)** Topographies show the results of comparisons between inversion effects (i.e., interaction effects): the first and the second images show that face inversion and body inversion lead to significantly different changes in activity over the previously reported areas when compared to house inversion. The third image shows that face and body inversion effects did not differ in a statistically significant way.

Inverted faces showed a significantly larger N170 component than upright faces over a large bilateral occipito-temporal cluster of 34 electrodes (*p* = 0.001) and a frontal cluster of 48 electrodes (*p* = 0.001). Similarly, inverted bodies showed a significantly larger N170 component than upright bodies over a right occipito-temporal cluster (22 electrodes; *p* = 0.008) and a left frontal cluster (23 electrodes; *p* = 0.008). No statistically significant differences were found between upright and inverted houses (Figure 4A).

The results also revealed significant interactions between face inversion and house inversion over a bilateral occipito-temporal cluster (29 electrodes; *p* = 0.008) and a frontal cluster (44 electrodes; *p* = 0.003), suggesting that face inversion generates an increase in N170 amplitude that is significantly higher than the increase generated by house inversion. Moreover, a statistically significant interaction between body inversion and house inversion was found over a right occipito-temporal cluster (19 electrodes; *p* = 0.025) and a left frontal cluster (20 electrodes; *p* = 0.017), indicating that the increase in N170 amplitude generated by body inversion was significantly higher than the increase generated by house inversion. Face inversion and body inversion showed no statistically significant differences (Figure 4B). In summary, ERPs indicate that physiological FIE and BIE show similar magnitude and that house stimuli do not show an inversion effect.

#### N170 Latency

The inversion * stimulus interaction effect resulted statistically significant (*F*_(2,110)_ = 11.458, *p* < 0.001). These results confirm inversion effects on N170 latency for faces (mean upright = 159 ms, mean inverted = 171 ms, *t*_(110)_ = 6.889, *p* < 0.001). This difference was smaller on houses (mean upright = 166 ms, mean inverted = 171 ms, *t*_(110)_ = 2.665, *p* = 0.009) and disappeared on bodies (mean upright = 165 ms, mean inverted = 165 ms, *t*_(110)_ = 0.197, *p* = 0.844). In summary, these results show that delayed N170 components were found for inverted (vs. upright) faces, houses, but not bodies.

### Induced Activity

**Figure 5:**
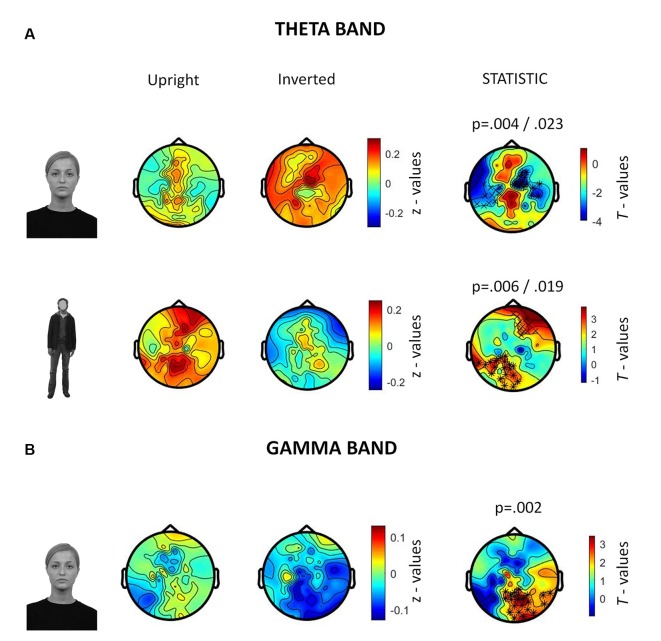
Summary of results related to the induced activity. All topographies were obtained by calculating the average power over the TOI for induced activity (i.e., 250–500 ms after stimulus onset). **(A)** Topographies show the activity induced by upright and inverted faces (first row) and bodies (second row) in the theta band (5–7 Hz). The third column of topographies shows clusters where statistically significant differences between upright and inverted stimuli were found by means of non-parametric cluster-based permutation tests. Increased theta synchronization is highlighted for inverted faces (vs. upright faces) over a right frontoparietal and a left parietal cluster, whereas upright bodies (vs. inverted bodies) induced a significantly stronger theta synchronization over a left-lateralized occipito-temporal cluster and a right prefrontal cluster. **(B)** Topographies representing gamma-band (28–45 Hz) activity induced by upright and inverted faces are shown. The statistical comparison highlighted a stronger gamma synchronization for upright faces (vs. inverted faces) over the right occipito-temporal cluster.

In the 250–500 ms time-window, a neurophysiological FIE showed that inverted faces, compared to upright faces, showed stronger theta synchronization in a right fronto-parietal cluster (14 electrodes; *p* = 0.004) and in a left parietal cluster (10 electrodes; *p* = 0.023; Figure 5A). Participants also showed a stronger theta synchronization induced by upright bodies (compared to inverted bodies) in a left-lateralized occipito-temporal cluster (19 electrodes; *p* = 0.006) and in a right prefrontal cluster (12 electrodes; *p* = 0.019).

The analysis of gamma-band activity showed stronger synchronization for upright faces than inverted faces (*p* = 0.002) over a right-lateralized occipito-temporal cluster of electrodes (21 electrodes; Figure 5B). No other inversion effects were statistically significant. In summary, gamma-band activity is reduced by face inversion only. FIE and BIE showed an opposite physiological pattern in theta: enhanced theta by face inversion and reduced theta by body inversion.

## Discussion

At the behavioral level, upright face and body perception are mediated by configural processing, which allows perceiving these stimuli as a *gestalt* (McKone and Yovel, 2009), rather than the sum of the individual parts. Configural processing does not mediate the perception of inverted faces and bodies, which relies on part-based mechanisms (Maurer et al., 2002; Reed et al., 2006); this is why performance on face and body perception tasks drops after stimulus inversion (i.e., the inversion effect; Yin, 1969; Reed et al., 2003) and this has been suggested to reflect the presence of configural mechanisms for the perception of both categories of visual stimuli (Rivolta et al., 2017; Bonemei et al., 2018). Given that object perception is not affected by stimulus inversion, it has been suggested that object processing is only mediated by part-based, and not by configural, mechanisms (Gauthier and Tarr, 2002; Maurer et al., 2002). In summary, behavioral evidence indicates that both face and body perception relies on configural processing. What remains unknown is whether the inversion effects for faces and bodies are mediated by similar neurophysiological activity. Our findings, in line with previous evidence (Watanabe et al., 2003; Stekelenburg and de Gelder, 2004), indicate that FIE and BIE are characterized by a larger N170 for inverted stimuli (Rossion and Gauthier, 2002); this was spread over the bilateral occipito-temporal region for faces, albeit more lateralized to the right hemisphere for bodies (see Gliga and Dehaene-Lambertz, 2005 for a similar finding). Results found in N170 latency reflect what can be intuitively observed from Figure 1: delayed N170 components were found for inverted (vs. upright) faces, houses, but not bodies. These results show that the N170 delay is not a consistent and replicable index of BIE, as opposed to what was found for FIE (Rossion et al., 2000). This is in line with previous evidence showing the absence of BIE on N170 latency, despite its presence on N170 amplitude (Minnebusch et al., 2010; Mohamed et al., 2011; Soria Bauser and Suchan, 2013).

Results on induced neural oscillations, however, revealed clear differences between FIE and BIE. Specifically, *face* inversion induced gamma-band desynchronization over occipito-temporal electrodes (developing to the right side) and synchronization in the theta band over bilateral frontoparietal regions, whereas *body* inversion induced a desynchronization in the theta band over left occipito-temporal and right prefrontal areas. Therefore, it is important to try to differentiate between the theta synchronization found for inverted faces, and that found for upright bodies: here it is important to point out that cerebral activity in the same frequency band may mediate completely different functions in different cortical (and sub-cortical) areas and in different time-windows (Başar, 1999).

### Gamma and Theta Correlates of Face Perception

Upright face perception, as compared to inverted face or object perception, is mediated by stronger gamma-band activity (Tallon-Baudry and Bertrand, 1999). Our results corroborate this finding and indicate that occipital gamma-band activity is a neurophysiological correlate of holistic face processing that, by definition, mediates upright face stimuli perception (Rodriguez et al., 1999; Anaki et al., 2007). The replication of previous findings in the gamma band demonstrates the reliability of the experimental paradigm and of the task we used, thus granting stronger reliability to our explanation of the novel results that we found in the theta band.

Activity in the theta band has been related to attention (Başar, 1999; Klimesch, 1999), and it has been specifically associated with feature-based attentional functions (i.e., attentional resources deployed to process single features of visual stimuli, not bound in a configuration; Harris et al., 2017). It is thus possible that theta-band synchronization associated with the FIE reflects an increase in attentional resources towards inverted faces (which may have been needed to recognize their orientation rapidly) since the holistic face processing is disrupted by face inversion (Maurer et al., 2002). Furthermore, the scalp regions showing the FIE in theta are compatible with areas involved in the dorsal frontoparietal attentional network, responsible for endogenous allocation and maintenance of visuospatial attention (Corbetta et al., 2002; Ptak, 2012; Lückmann et al., 2014; Ptak et al., 2017). Even though it is known that upright faces automatically capture attention through a stimulus-driven/bottom-up mechanism (Sato and Kawahara, 2015), this is not the case for inverted faces (Langton et al., 2008; Tomonaga and Imura, 2009; Sato and Kawahara, 2015; Ariga and Arihara, 2018). Hence, it is likely that an explicit (top-down) attentional effort is required to process facial features that cannot be bound in a configuration since holistic processing is disrupted by inversion. This deployment of attentional resources may be what triggers the increased theta activation in the frontoparietal network.

### Gamma and Theta Correlates of Body Perception

Contrary to what we observed for faces, upright (as compared to inverted) body perception induced greater theta synchronization over the left occipito-temporal and right prefrontal areas, whereas no significant differences were found in the gamma band. These results may suggest that (upright) body processing appears to be more related to the first stages of configural processing (i.e., first-order spatial information and structural information; Maurer et al., 2002; Reed et al., 2006) and feature-based processing. By contrast, differences that emerged during face processing are more related to the holistic processing of the stimulus, so that inverted faces require greater cognitive effort to be recognized. This interpretation is supported by findings showing that holistic processing may not be involved in the perception of human body forms (Soria Bauser et al., 2011, 2015; Soria Bauser and Suchan, 2013). Indeed, it has never been demonstrated that holistic processing and second-order spatial information processing are part of configural body processing (Minnebusch and Daum, 2009), whereas first-order spatial information and structural information have been shown to be involved in it, when considering both behavioral (Reed et al., 2006) and neuroimaging results (Brandman and Yovel, 2014).

Additionally, the occipito-temporal synchronization showed clear lateralization to the left hemisphere. The literature has reported right lateralization for ERPs involved in body processing (for a review, see Gliga and Dehaene-Lambertz, 2005; de Gelder et al., 2015), similar to what we found for the N170. However, no previous studies have investigated oscillations in body processing. Considering that induced activity excludes, for the most part, evoked (time-locked) oscillations (i.e., activity related to ERPs), the left occipito-temporal induced synchronization represents a novel finding: while the early stages of body processing are primarily lateralized to the right hemisphere, a later (greater than 250 ms post-stimulus) stage of processing seems to be left-lateralized and consists of induced oscillations in theta range. This result may imply the involvement of bilateral occipito-temporal cortices in different stages of body processing, constituting an important difference with the overall right-lateralization of face processing. This new result requires corroboration in future research.

Upright and inverted houses did not show statistically significant differences in any of the considered frequency bands. This result confirms that this class of stimuli is much less subject to inversion effects since their perception relies mostly on part-based processing (Negrini et al., 2017). The lack of inversion effect in houses also confirms that the results we found are indeed specific for faces and bodies.

### Conclusions and Future Directions

In this study we found new evidence that the neurophysiological mechanisms mediating face and body inversion effects have important differences (Reed et al., 2006; Soria Bauser et al., 2011, 2015; Soria Bauser and Suchan, 2013): our results indicated the involvement of feature-binding processes for faces (i.e., occipital gamma activity). In addition, stimulus inversion can disrupt these processes and seems to require feature-based processing (i.e., theta frontoparietal activity). By contrast, the BIE appears to be less related to holistic processing and more to the first stages of configural processing.

While previous studies have investigated the causal relationship between neural markers of the BIE and the structural encoding process, which is involved, the present study is more explorative and correlational in nature, which however represents a limitation for most EEG/MEG and functional magnetic resonance imaging (fMRI) research. In other terms, we could indicate neurophysiological correlates of stimuli inversion, but without evidence of a causal relationship between them. However, to the best of our knowledge, no previous studies have investigated the oscillatory aspects of neural processes involved in body processing and, a fortiori, in the body inversion effect. A possible way to establish a causal correlation between behavior and neural oscillations might be, for instance, the adoption of transcranial alternate current stimulation (tACS; Gonzalez-Perez et al., 2019). Future work should also directly assess holistic processing by using different behavioral tasks such as the composite face task (Rossion, 2013).

## Data Availability Statement

The datasets generated for this study are available on request to the corresponding author.

## Ethics Statement

The studies involving human participants were reviewed and approved by the local Ethics Committee of the University of East London (UEL) and was conducted in accordance with the ethical standards laid out in the 1964 Declaration of Helsinki. The patients/participants provided their written informed consent to participate in this study.

## Author Contributions

FB, PR and DR designed the experiment. FB and SB acquired the data. FB, IP and SP analyzed the data. FB, IP, PR and DR wrote the manuscript.

## Conflict of Interest

The authors declare that the research was conducted in the absence of any commercial or financial relationships that could be construed as a potential conflict of interest.

## References

[B1] Adobe Systems, Inc (2011). Adobe photoshop Cs5. Methods.

[B2] Aftanas L. I. Varlamov A. A. Pavlov S. V. Makhnev V. P. Reva N. V. (2001). Affective picture processing: event-related synchronization within individually defined human theta band is modulated by valence dimension. Neurosci. Lett. 303, 115–118. 10.1016/s0304-3940(01)01703-7 11311506

[B3] Aftanas L. I. Varlamov A. A. Pavlov S. V. Makhnev V. P. Reva N. V. (2002). Time-dependent cortical asymmetries induced by emotional arousal: EEG analysis of event-related synchronization and desynchronization in individually defined frequency bands. Int. J. Psychophysiol. 44, 67–82. 10.1016/s0167-8760(01)00194-5 11852158

[B4] Anaki D. Zion-Golumbic E. Bentin S. (2007). Electrophysiological neural mechanisms for detection, configural analysis and recognition of faces. NeuroImage 37, 1407–1416. 10.1016/j.neuroimage.2007.05.054 17689102

[B5] Ariga A. Arihara K. (2018). Attentional capture by spatiotemporally task-irrelevant faces: supportive evidence for Sato and Kawahara (2015). Psychol. Res. 82, 859–865. 10.1007/s00426-017-0869-3 28455652

[B6] Balconi M. Lucchiari C. (2006). EEG correlates (event-related desynchronization) of emotional face elaboration: a temporal analysis. Neurosci. Lett. 392, 118–123. 10.1016/j.neulet.2005.09.004 16202519

[B7] Başar E. (1999). Brain Function and Oscillations: Volume II. Integrative Brain Function. Neurophysiology and Cognitive Processes. Heidelberg: Springer Series in Synergetics.

[B8] Başar E. (2012). Multiple oscillations and phase locking in human γ responses: an essay in search of eigenvalues. NeuroQuantology 4:603 10.14704/nq.2012.10.4.603

[B9] Başar E. Güntekin B. Öniz A. (2006). Chapter 4 Principles of oscillatory brain dynamics and a treatise of recognition of faces and facial expressions. Prog. Brain Res. 159, 43–62. 10.1016/s0079-6123(06)59004-1 17071223

[B10] Bentin S. Allison T. Puce A. Perez E. McCarthy G. (1996). Electrophysiological studies of face perception in humans. J. Cogn. Neurosci. 8, 551–565. 10.1162/jocn.1996.8.6.551 20740065 PMC2927138

[B11] Bentin S. Deouell L. Y. Soroker N. (1999). Selective visual streaming in face recognition: evidence from developmental prosopagnosia. Neuroreport 10, 823–827. 10.1097/00001756-199903170-00029 10208555

[B12] Bonemei R. Costantino A. I. Battistel I. Rivolta D. (2018). The perception of (naked only) bodies and faceless heads relies on holistic processing: evidence from the inversion effect. Br. J. Psychol. 109, 232–243. 10.1111/bjop.12271 28940474

[B13] Brandman T. Yovel G. (2014). Bodies are represented as wholes rather than their sum of parts in the occipital-temporal cortex. Cereb. Cortex 26, 530–543. 10.1093/cercor/bhu205 25217470

[B14] Cohen M. X. Donner T. H. (2013). Midfrontal conflict-related theta-band power reflects neural oscillations that predict behavior. J. Neurophysiol. 110, 2752–2763. 10.1152/jn.00479.2013 24068756

[B15] Corbetta M. Kincade J. M. Shulman G. L. (2002). Neural systems for visual orienting and their relationships to spatial working memory. J. Cogn. Neurosci. 14, 508–523. 10.1162/089892902317362029 11970810

[B16] David O. Kilner J. M. Friston K. J. (2006). Mechanisms of evoked and induced responses in MEG/EEG. NeuroImage 31, 1580–1591. 10.1016/j.neuroimage.2006.02.034 16632378

[B18] de Gelder B. de Borst A. W. Watson R. (2015). The perception of emotion in body expressions. Wiley Interdiscip. Rev. Cogn. Sci. 6, 149–158. 10.1002/wcs.1335 26263069

[B17] de Gelder B. Van den Stock J. (2011). The bodily expressive action stimulus test (BEAST). Construction and validation of a stimulus basis for measuring perception of whole body expression of emotions. Front. Psychol. 2:181. 10.3389/fpsyg.2011.00181 21886632 PMC3152787

[B19] Delorme A. Makeig S. (2004). EEGLAB: an open source toolbox for analysis of single-trial EEG dynamics including independent component analysis. J. Neurosci. Methods 134, 9–21. 10.1016/j.jneumeth.2003.10.009 15102499

[B20] Dien J. (1998). Issues in the application of the average reference: review, critiques, and recommendations. Behav. Res. Methods Instrum. Comput. 30, 34–43. 10.3758/bf03209414

[B21] Dobel C. Junghöfer M. Gruber T. (2011). The role of γ-band activity in the representation of faces: reduced activity in the fusiform face area in congenital prosopagnosia. PLoS One 6:e19550. 10.1371/journal.pone.0019550 21573175 PMC3088687

[B22] Donner T. H. Siegel M. (2011). A framework for local cortical oscillation patterns. Trends Cogn. Sci. 15, 191–199. 10.1016/j.tics.2011.03.007 21481630

[B23] Gao Z. Goldstein A. Harpaz Y. Hansel M. Zion-Golumbic E. Bentin S. (2012). A magnetoencephalographic study of face processing: M170, γ-band oscillations and source localization. Hum. Brain Mapp. 34, 1783–1795. 10.1002/hbm.22028 22422432 PMC3382029

[B24] Gauthier I. Tarr M. J. (2002). Unraveling mechanisms for expert object recognition: bridging brain activity and behavior. J. Exp. Psychol. Hum. Percept. Perform. 28, 431–446. 10.1037/0096-1523.28.2.431 11999864

[B25] Gliga T. Dehaene-Lambertz G. (2005). Structural encoding of body and face in human infants and adults. J. Cogn. Neurosci. 17, 1328–1340. 10.1162/0898929055002481 16197687

[B26] Gonzalez-Perez M. Wakui E. Thoma V. Nitsche M. A. Rivolta D. (2019). Transcranial alternating current stimulation (tACS) at 40 Hz enhances face and object perception. Neuropsychologia 135:107237 10.1016/j.neuropsychologia.2019.107237 31655161

[B27] Grandchamp R. Delorme A. (2011). Single-trial normalization for event-related spectral decomposition reduces sensitivity to noisy trials. Front. Psychol. 2:236. 10.3389/fpsyg.2011.00236 21994498 PMC3183439

[B90] Grent-t’-Jong T. Rivolta D. Sauer A. Grube M. Singer W. Wibral M.. (2016). MEG-measured visually induced gamma-band oscillations in chronic schizophrenia: Evidence for impaired generation of rhythmic activity in ventral stream regions. Schizophrenia research 176, 177–185. 10.1016/j.schres.2016.06.003 27349815

[B28] Güntekin B. Başar E. (2009). Facial affect manifested by multiple oscillations. Int. J. Psychophysiol. 71, 31–36. 10.1016/j.ijpsycho.2008.07.019 18725252

[B29] Güntekin B. Başar E. (2014). A review of brain oscillations in perception of faces and emotional pictures. Neuropsychologia 58, 33–51. 10.1016/j.neuropsychologia.2014.03.014 24709570

[B91] Grützner C. Wibral M. Sun L. Rivolta D. Singer W. Maurer K.. (2013). Deficits in high- (>60 Hz) gamma-band oscillations during visual processing in schizophrenia. Front. Hum. Neurosci. 7, 88. 10.3389/fnhum.2013.00088 23532620 PMC3607810

[B30] Harris A. M. Dux P. E. Jones C. N. Mattingley J. B. (2017). Distinct roles of theta and α oscillations in the involuntary capture of goal-directed attention. NeuroImage 152, 171–183. 10.1016/j.neuroimage.2017.03.008 28274832

[B32] Herrmann C. Rach S. Vosskuhl J. Strüber D. (2014). Time-frequency analysis of event-related potentials: a brief tutorial. Brain Topogr. 27, 438–450. 10.1007/s10548-013-0327-5 24194116

[B33] Iacoboni M. Molnar-Szakacs I. Gallese V. Buccino G. Mazziotta J. C. Rizzolatti G. (2005). Grasping the intentions of others with one’s own mirror neuron system. PLoS Biol. 3:e79. 10.1371/journal.pbio.0030079 15736981 PMC1044835

[B34] Klimesch W. (1999). EEG α and theta oscillations reflect cognitive and memory performance: a review and analysis. Brain Res. Rev. 29, 169–195. 10.1016/s0165-0173(98)00056-3 10209231

[B35] Knyazev G. G. Slobodskoj-Plusnin J. Y. Bocharov A. V. (2009). Event-related delta and theta synchronization during explicit and implicit emotion processing. Neuroscience 164, 1588–1600. 10.1016/j.neuroscience.2009.09.057 19796666

[B36] Lachaux J. P. George N. Tallon-Baudry C. Martinerie J. Hugueville L. Minotti L.. (2005). The many faces of the γ band response to complex visual stimuli. NeuroImage 25, 491–501. 10.1016/j.neuroimage.2004.11.052 15784428

[B37] Langner O. Dotsch R. Bijlstra G. Wigboldus D. H. J. Hawk S. T. van Knippenberg A. (2010). Presentation and validation of the radboud faces database. Cogn. Emot. 24, 1377–1388. 10.1080/02699930903485076

[B38] Langton S. R. H. Law A. S. Burton A. M. Schweinberger S. R. (2008). Attention capture by faces. Cognition 107, 330–342. 10.1016/j.cognition.2007.07.012 17767926

[B39] Lopes da Silva F. (1992). “The rhythmic slow activity (theta) of the limbic cortex: an oscillation in search of a function,” in Induced Rhythms in the Brain, eds Başar E. Bullock T. H. (Boston: Birkhauser), 83–102.

[B40] Lückmann H. C. Jacobs H. I. L. Sack A. T. (2014). The cross-functional role of frontoparietal regions in cognition: internal attention as the overarching mechanism. Prog. Neurobiol. 116, 66–86. 10.1016/j.pneurobio.2014.02.002 24530293

[B41] Maris E. (2012). Statistical testing in electrophysiological studies. Psychophysiology 49, 549–565. 10.1111/j.1469-8986.2011.01320.x 22176204

[B42] Maris E. Oostenveld R. (2007). Nonparametric statistical testing of EEG- and MEG-data. J. Neurosci. Methods 164, 177–190. 10.1016/j.jneumeth.2007.03.024 17517438

[B43] Mathworks, Inc (2016). MATLAB (R2016a). Natick, MA: The MathWorks Inc.

[B44] Matsuzaki N. Schwarzlose R. F. Nishida M. Ofen N. Asano E. (2015). Upright face-preferential high-γ responses in lower-order visual areas: evidence from intracranial recordings in children. NeuroImage 109, 249–259. 10.1016/j.neuroimage.2015.01.015 25579446 PMC4340724

[B45] Maurer D. Grand R. L. Mondloch C. J. (2002). The many faces of configural processing. Trends Cogn. Sci. 6, 255–260. 10.1016/s1364-6613(02)01903-4 12039607

[B46] McKone E. Yovel G. (2009). Why does picture-plane inversion sometimes dissociate perception of features and spacing in faces and sometimes not? Toward a new theory of holistic processing. Psychon. Bull. Rev. 16, 778–797. 10.3758/pbr.16.5.778 19815781

[B47] Minnebusch D. A. Daum I. (2009). Neuropsychological mechanisms of visual face and body perception. Neurosci. Biobehav. Rev. 33, 1133–1144. 10.1016/j.neubiorev.2009.05.008 19500617

[B48] Minnebusch D. A. Keune P. M. Suchan B. Daum I. (2010). Gradual inversion affects the processing of human body shapes. NeuroImage 49, 2746–2755. 10.1016/j.neuroimage.2009.10.046 19853043

[B49] Mohamed T. N. Neumann M. F. Schweinberger S. R. (2011). Combined effects of attention and inversion on event-related potentials to human bodies and faces. Cogn. Neurosci. 2, 138–146. 10.1080/17588928.2011.597848 24168528

[B50] Monti C. Sozzi M. Bossi F. Corbo M. Rivolta D. (2020). Atypical holistic processing of facial identity and expression in a case of acquired prosopagnosia. Cogn. Neuropsychol. [Epub ahead of print]. 10.1080/02643294.2020.1718071 31983272

[B51] Moratti S. Méndez-Bértolo C. Del-Pozo F. Strange B. A. (2014). Dynamic γ frequency feedback coupling between higher and lower order visual cortices underlies perceptual completion in humans. NeuroImage 86, 470–479. 10.1016/j.neuroimage.2013.10.037 24185019

[B52] Negrini M. Brkić D. Pizzamiglio S. Premoli I. Rivolta D. (2017). Neurophysiological correlates of featural and spacing processing for face and non-face stimuli. Front. Psychol. 8:333. 10.3389/fpsyg.2017.00333 28348535 PMC5346548

[B54] Oostenveld R. Fries P. Maris E. Schoffelen J. M. (2011). FieldTrip: open source software for advanced analysis of MEG, EEG, and invasive electrophysiological data. Comput. Intell. Neurosci. 2011:156869. 10.1155/2011/156869 21253357 PMC3021840

[B55] Premoli I. Bergmann T. O. Fecchio M. Rosanova M. Biondi A. Belardinelli P.. (2017). The impact of GABAergic drugs on TMS-induced brain oscillations in human motor cortex. NeuroImage 163, 1–12. 10.1016/j.neuroimage.2017.09.023 28917695

[B56] Ptak R. (2012). The frontoparietal attention network of the human brain: action, saliency, and a priority map of the environment. Neuroscientist 18, 502–515. 10.1177/1073858411409051 21636849

[B57] Ptak R. Schnider A. Fellrath J. (2017). The dorsal frontoparietal network: a core system for emulated action. Trends Cogn. Sci. 21, 589–599. 10.1016/j.tics.2017.05.002 28578977

[B58] Reed C. L. Stone V. E. Bozova S. Tanaka J. (2003). The body-inversion effect. Psychol. Sci. 14, 302–308. 10.1111/1467-9280.14431 12807401

[B59] Reed C. L. Stone V. E. Grubb J. D. McGoldrick J. E. (2006). Turning configural processing upside down: part and whole body postures. J. Exp. Psychol. Hum. Percept. Perform. 32, 73–87. 10.1037/0096-1523.32.1.73 16478327

[B60] Rivolta D. Castellanos N. P. Stawowsky C. Helbling S. Wibral M. Grützner C.. (2014). Source-reconstruction of event-related fields reveals hyperfunction and hypofunction of cortical circuits in antipsychotic-naive, first-episode schizophrenia patients during Mooney face processing. J. Neurosci. 34, 5909–5917. 10.1523/JNEUROSCI.3752-13.2014 24760850 PMC6608292

[B61] Rivolta D. Palermo R. Schmalzl L. Williams M. A. (2012). Investigating the features of the M170 in congenital prosopagnosia. Front. Hum. Neurosci. 6:45. 10.3389/fnhum.2012.00045 22416228 PMC3298857

[B88] Rivolta D. Heidegger T. Scheller B. Sauer A. Schaum M. Birkner K.. (2015). Ketamine dysregulates the amplitude and connectivity of high-frequency oscillations in cortical–subcortical networks in humans: Evidence from resting-state magnetoencephalography-recordings. Schizophrenia bulletin 41, 1105–1114. 10.1093/schbul/sbv051 25987642 PMC4535642

[B89] Rivolta D. Lawsona R. P. Palermo R. (2017). More than just a problem with faces: Altered body perception in a group of congenital prosopagnosics. Quarterly Journal of Experimental Psychology 70, 276–286. 10.10.1080/17470218.2016.1174277 27049475

[B62] Robbins R. McKone E. (2007). No face-like processing for objects-of-expertise in three behavioural tasks. Cognition 103, 34–79. 10.1016/j.cognition.2006.02.008 16616910

[B63] Rodriguez E. George N. Lachaux J. P. Martinerie J. Renault B. Varela F. J. (1999). Perception’s shadow: long-distance synchronization of human brain activity. Nature 397, 430–433. 10.1038/17120 9989408

[B64] Rossion B. (2013). The composite face illusion: a whole window into our understanding of holistic face perception. Vis. Cogn. 21, 139–253. 10.1080/13506285.2013.772929

[B65] Rossion B. Gauthier I. (2002). How does the brain process upright and inverted faces? Behav. Cogn. Neurosci. Rev. 1, 63–75. 10.1177/1534582302001001004 17715586

[B66] Rossion B. Gauthier I. Tarr M. J. Despland P. Bruyer R. Linotte S.. (2000). The N170 occipito-temporal component is delayed and enhanced to inverted faces but not to inverted objects: an electrophysiological account of face-specific processes in the human brain. Neuroreport 11, 69–74. 10.1097/00001756-200001170-00014 10683832

[B67] Sato S. Kawahara J. I. (2015). Attentional capture by completely task-irrelevant faces. Psychol. Res. 79, 523–533. 10.1007/s00426-014-0599-8 25030814

[B68] Sato W. Kochiyama T. Uono S. Matsuda K. Usui K. Inoue Y.. (2014). Rapid, high-frequency, and theta-coupled γ oscillations in the inferior occipital gyrus during face processing. Cortex 60, 52–68. 10.1016/j.cortex.2014.02.024 24745564

[B69] Sato W. Kochiyama T. Uono S. Matsuda K. Usui K. Usui N.. (2017). Bidirectional electric communication between the inferior occipital gyrus and the amygdala during face processing. Hum. Brain Mapp. 38, 4511–4524. 10.1002/hbm.23678 28573679 PMC6867177

[B70] Schneider W. Eschman A. Zuccolotto A. (2007). E-Prime® 2.0. Pittsburg: Psychological Software Inc.

[B71] Singer W. Gray C. M. (1995). Visual feature integration and the temporal correlation hypothesis. Annu. Rev. Neurosci. 18, 555–586. 10.1146/annurev.ne.18.030195.003011 7605074

[B73] Soria Bauser D. A. Schriewer E. Suchan B. (2015). Dissociation between the behavioural and electrophysiological effects of the face and body composite illusions. Br. J. Psychol. 106, 414–432. 10.1111/bjop.12101 25330089

[B72] Soria Bauser D. A. Suchan B. (2013). Behavioral and electrophysiological correlates of intact and scrambled body perception. Clin. Neurophysiol. 124, 686–696. 10.1016/j.clinph.2012.09.030 23375380

[B74] Soria Bauser D. A. Suchan B. Daum I. (2011). Differences between perception of human faces and body shapes: evidence from the composite illusion. Vision Res. 51, 195–202. 10.1016/j.visres.2010.11.007 21093471

[B75] Stekelenburg J. J. de Gelder B. (2004). The neural correlates of perceiving human bodies: an ERP study on the body-inversion effect. Cogn. Neurosci. Neuropsychol. 15, 9–12. 10.1097/00001756-200404090-00007 15073513

[B76] Tallon-Baudry C. Bertrand O. (1999). Oscillatory γ activity in humans and its role in object representation. Trends Cogn. Sci. 3, 151–162. 10.1016/s1364-6613(99)01299-1 10322469

[B77] Tomonaga M. Imura T. (2009). Faces capture the visuospatial attention of chimpanzees (Pan troglodytes): evidence from a cueing experiment. Front. Zool. 6:14. 10.1186/1742-9994-6-14 19627571 PMC2718869

[B78] Tucker D. M. (1993). Spatial sampling of head electrical fields: the geodesic sensor net. Electroencephalogr. Clin. Neurophysiol. 87, 154–163. 10.1016/0013-4694(93)90121-b 7691542

[B79] Uhlhaas P. J. Singer W. (2010). Abnormal neural oscillations and synchrony in schizophrenia. Nat. Rev. Neurosci. 11, 100–113. 10.1038/nrn2774 20087360

[B80] Uono S. Sato W. Kochiyama T. Kubota Y. Sawada R. Yoshimura S.. (2017). Time course of γ-band oscillation associated with face processing in the inferior occipital gyrus and fusiform gyrus: a combined fMRI and MEG study. Hum. Brain Mapp. 38, 2067–2079. 10.1002/hbm.23505 28029717 PMC6867163

[B81] Valentine T. (1988). Upside-down faces: a review of the effect of inversion upon face recognition. Br. J. Psychol. 79, 471–491. 10.1111/j.2044-8295.1988.tb02747.x 3061544

[B82] Watanabe S. Kakigi R. Puce A. (2003). The spatiotemporal dynamics of the face inversion effect: a magneto- and electro-encephalographic study. Neuroscience 116, 879–895. 10.1016/s0306-4522(02)00752-2 12573727

[B83] Willenbockel V. Sadr J. Fiset D. Horne G. O. Gosselin F. Tanaka J. W. (2010). Controlling low-level image properties: the SHINE toolbox. Behav. Res. Methods 42, 671–684. 10.3758/BRM.42.3.671 20805589

[B84] Yin R. K. (1969). Looking at upside-down faces. J. Exp. Psychol. 81, 141–145. 10.1037/h0027474

[B85] Zhang D. Wang L. Luo Y. Luo Y. (2012). Individual differences in detecting rapidly presented fearful faces. PLoS One 7:e49517. 10.1371/journal.pone.0049517 23166693 PMC3498139

[B86] Zion-Golumbic E. Bentin S. (2007). Dissociated neural mechanisms for face detection and configural encoding: evidence from N170 and induced γ-band oscillation effects. Cereb. Cortex 17, 1741–1749. 10.1093/cercor/bhl100 17062635

[B87] Zion-Golumbic E. Golan T. Anaki D. Bentin S. (2008). Human face preference in γ-frequency EEG activity. NeuroImage 39, 1980–1987. 10.1016/j.neuroimage.2007.10.025 18083564 PMC2268116

